# Metformin ameliorates sepsis-induced brain injury by inhibiting apoptosis, oxidative stress and neuroinflammation via the PI3K/Akt signaling pathway

**DOI:** 10.18632/oncotarget.20105

**Published:** 2017-08-10

**Authors:** Guangming Tang, Huiyun Yang, Jing Chen, Mengrao Shi, Lingqing Ge, Xuhua Ge, Guoji Zhu

**Affiliations:** ^1^ Neonate Department, Soochow University Affiliated Children's Hospital, Suzhou, Jiangsu, P.R. China; ^2^ Department of Internal Medicine, Soochow University Affiliated Children's Hospital, Suzhou, Jiangsu, P.R. China; ^3^ Department of General Medicine, Yangpu Hospital Tongji University School of Medicine, Shanghai, P.R. C

**Keywords:** sepsis, metformin, inflammation, oxidative stress, cognitive impairment

## Abstract

Sepsis-induced brain injuries increase mortality, morbidity, cognitive impairment and lack of effective therapeutic treatment. Previous studies have suggested that metformin provides neuroprotective effects against ischemia, brain trauma and other brain damage, but whether metformin protects a septic brain remains unknown. Thus, the aim of this study is to investigate the possible effects and the mechanism of metformin against septic brain damage using the cecal ligation and puncture (CLP) model. Mice were randomly divided into five groups: the Sham group, CLP group, CLP+ Met group, CLP+ vehicle group and CLP+ Met+ LY group. The survival percentage and brain water content were examined, and the Morris water maze was conducted to determine the protective effect of metformin. Neuronal apoptosis in the cerebral cortex, striatum and hippocampus was examined using TUNEL assay and immunohistochemistry, and western blot was applied to measure the expression of p-Akt. The results indicate that metformin can increase survival percentage, decrease brain edema, preserve the blood-brain barrier (BBB) and improve cognitive function. Metformin also reduced the neuronal apoptosis induced by sepsis and increased the phosphorylation of Akt. However, the protective effect of metformin can be reversed by LY294002, a PI3K inhibitor. In summary, our results demonstrate that metformin can exert a neuroprotective effect by activating the PI3K/Akt signaling pathway.

## INTRODUCTION

Sepsis and septic shock are characterized by an overwhelming systemic inflammatory response and are complicated by the dysfunction of various organs, especially the brain [1]. During sepsis, patients suffer from brain dysfunction, such as an impairment of cerebral perfusion, the onset of the neuroinflammatory process, an alteration of the blood-brain barrier (BBB) and the passage of neurotoxic substances [2]. Furthermore, sepsis is associated with increased mortality, morbidity and cognitive impairment. Although our understanding of sepsis is advanced, the treatment of sepsis is still lacking in efficacy [3]. Thus, further investigation for the treatment of sepsis is of great importance.

In the pathogenesis of sepsis, three inflammatory hallmark cytokines—Interleukin-6 (IL-6), Interleukin-1β (IL-1β), and tumor necrosis factor-α (TNF-α)—are produced by monocytes and neutrophils [4]. These inflammatory cytokines invade, which leads to a systemic inflammatory response and brain injury. Further study proves that interleukin-3 (IL-3) amplifies acute inflammation and plays a critical role in the emergency myelopoiesis of sepsis [5]. Sepsis associated encephalopathy (SAE) develops in many patients with sepsis, and the changes in the cerebral microvasculature and breakdown of BBB reportedly contribute to SAE [6]. In addition to inflammatory toxicity, oxidative stress and neuronal apoptosis contribute to brain injuries resulting from sepsis. It has been reported that as reactive oxygen species (ROS) are overproduced in an animal model of sepsis [7], a pharmacological way to attenuate brain oxidative stress could perhaps exert a neuroprotective effect [8].

Cell apoptosis, which involves the phosphatidylinositol-3-kinase (PI3K)/Akt pathway, is critical in neuronal death associated with sepsis [9]. In addition, the PI3K/Akt pathway is firmly involved in cerebral ischemia and intracerebral hemorrhage [10, 11]. However, although the evidence suggests that preventing cell apoptosis could improve the survival rate in animal models with sepsis [12], it is also proposed that a pharmacological treatment of LY294002, a PI3K inhibitor, could reverse the existing protective effect of myocardial injury due to sepsis [13].

Metformin, a clinical drug, is widely used in the treatment of type 2 diabetes and metabolic syndrome because its enhance insulin sensitivity and decreases high blood sugar [14]. A previous study found that metformin exerts a cardio-protective effect in lipopolysaccharide-induced sepsis via the suppression of toll-like receptor 4 activity in the heart [15]. Studies further suggest that metformin has a direct anti-inflammatory action and can ameliorate oxidative stress [16, 17]. In a liver ischemic model, metformin protected the liver from the effects of anti-oxidation effect and anti-inflammation [18]. Thus, it is possible to use metformin in the treatment of sepsis to protect patients from brain injury.

The aim of this study is to investigate the effect of metformin in sepsis and the underlying mechanism of the protective effect using the cecal ligation and puncture (CLP) model. We hypothesize that in the CLP model, metformin ameliorates the inflammatory response, decreases oxidative stress and promotes the phosphorylation of Akt, which further inhibits the neuronal apoptosis in the cerebral cortex, striatum and hippocampus.

## RESULTS

### Metformin increased the survival percentage following CLP

**Figure 1:**
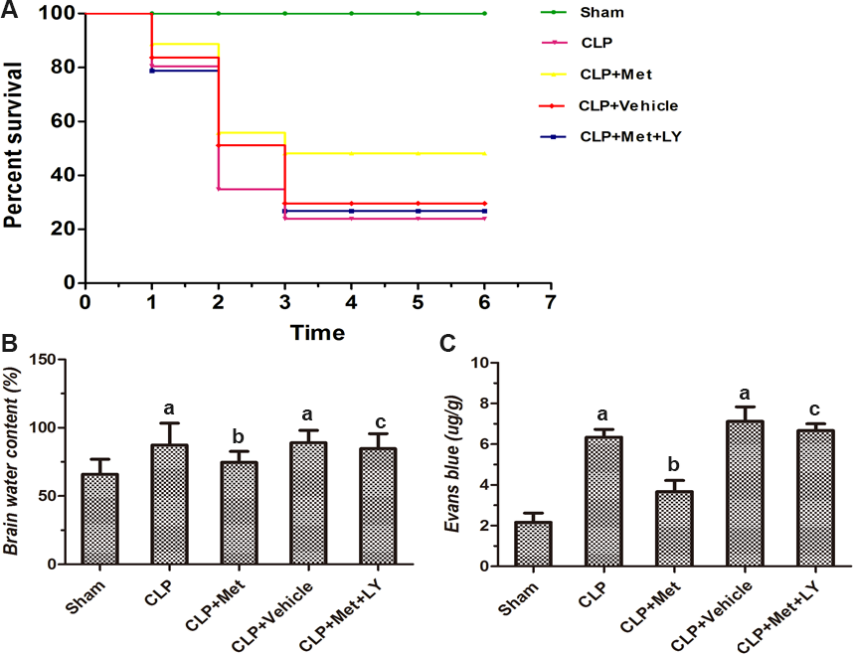
Effect of metformin on the 7-day survival percentage and brain edema after CLP surgery (**A**) Diagram shows the survival percentage of each group (*n* = 30 in each group). (**B**) Effect of metformin on brain water content for each group (*n* = 6 in each group). (**C**) Effect of metformin on blood-brain barrier (BBB) integrity; BBB integrity is presented as the amount of Evans blue extraction (*n* = 6 in each group); data are presented as the mean ± S.E.M.; (A) *P* < 0.05 relative to the Sham group; (B) *P* < 0.05 relative to the CLP group; (C) *P* < 0.05 relative to the CLP+ Met group.

To evaluate the effect of metformin on septic mice, we measured the survival percentage. The 7-day survival percentage in the Sham group was 100%. However, after CLP surgery, the survival percentage decreased dramatically. At day 7, the survival percentage of the CLP group dropped to 24% compared with the Sham group (*P <* 0.05, Figure 1A). With the administration of metformin, the survival percentage increased significantly to 50% compared with the CLP group (*P* < 0.05, Figure 1A). However, metformin had no effect on the CLP+ vehicle group. Moreover, the administration of LY decreased the survival percentage to 26% compared with the CLP+ Met group (*P <* 0.05, Figure 1A).

### Metformin attenuated cerebral edema and preserved BBB integrity following CLP

A statistical analysis revealed that septic mice exhibited a significant increase in brain water content and EB values compared with the CLP group (*P <* 0.05, Figure 1B–1C). Furthermore, although the administration of metformin can markedly alleviate cerebral edema and BBB permeability after CLP (*P <* 0.05, Figure 1B–1C), the protective effect of metformin was eliminated by the administration of LY in the CLP+ Met+ LY group (*P <* 0.05, Figure 1B–1C).

### Metformin decreased the pro-inflammatory cytokines following CLP

**Figure 2:**
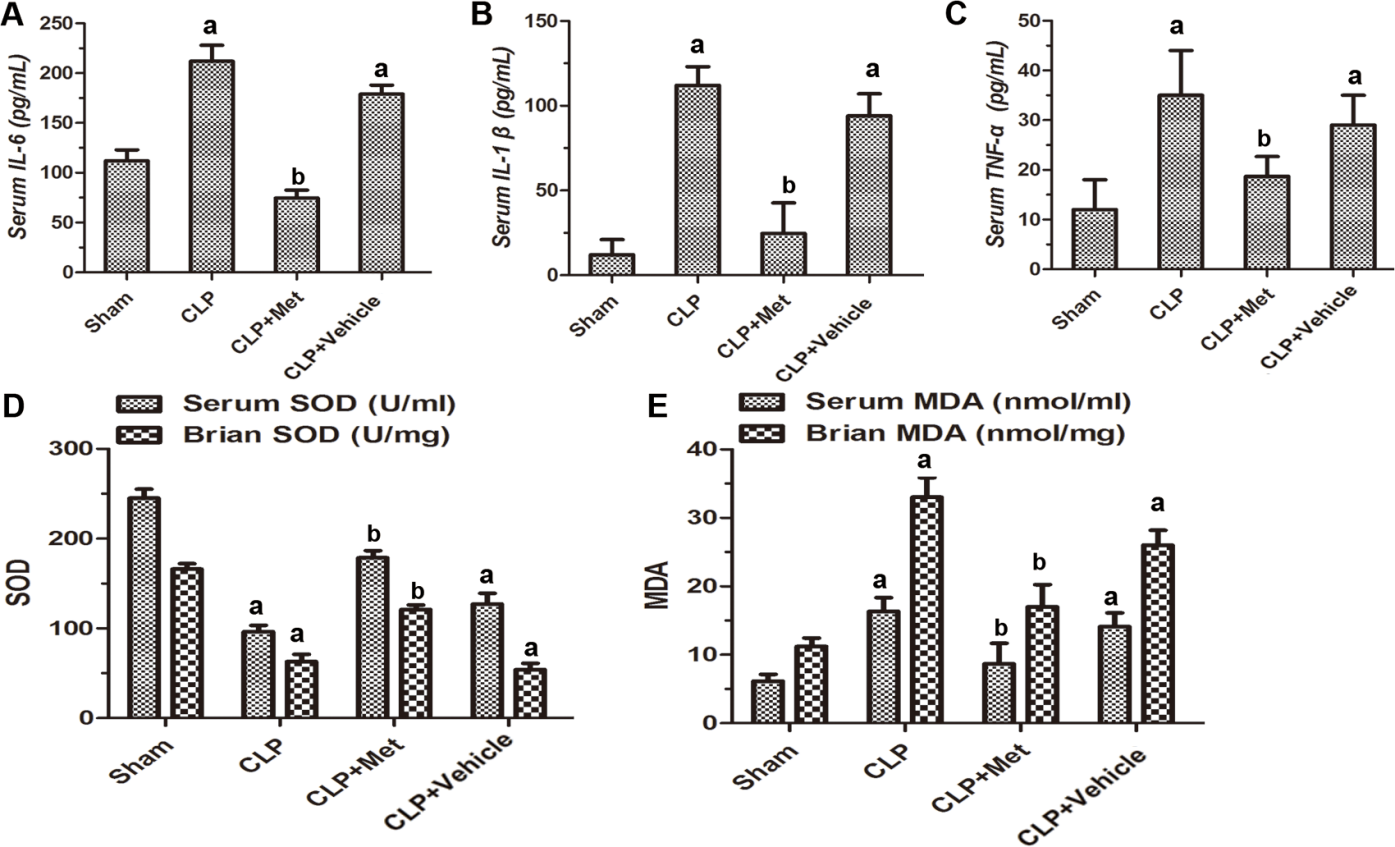
Effect of metformin on the levels of inflammatory cytokines and oxidative stress (**A**) Levels of IL-6 in the serum. (**B**) Levels of IL-1β in the serum. (**C**) Levels of TNF-α in the serum. (**D**) Activities of SOD in the serum and the brain. (**E**) Levels of MDA in the serum and the brain; data are presented as the mean ± S.E.M (*n* = 6); (A) *P* < 0.05 relative to the Sham group; (B) *P* < 0.05 relative to the CLP group.

We then measured the anti-inflammatory effect of metformin on septic mice. Our results revealed that the levels of serum IL-6, IL-1β and TNF-α dramatically increased after CLP (*P <* 0.05, Figure 2A–2C). With metformin treatment, the levels of serum IL-6, IL-1β and TNF-α were significantly decreased compared with the CLP group (*P <* 0.05, Figure 2A–2C). However, the treatment using normal saline to replace the metformin had no effect on CLP induced pro-inflammatory cytokines levels (*P <* 0.05, Figure 2A–2C).

### Metformin decreased oxidative stress following CLP

To study the effect of metformin on oxidative stress, we examined the activities of SOD and the levels of MDA. In the CLP group, the activities of SOD in serum and brain tissue were significantly decreased compared with those of the Sham group (*P <* 0.05, Figure 2D–2E), which was consistent with the treatment using normal saline. However, the administration of metformin resulted in remarkable increases in the activities of SOD (*P <* 0.05, Figure 2D–2E). In addition, metformin treatment decreased the MDA levels in the CLP+ Met group compared with those in the CLP group (*P <* 0.05, Figure 2D–2E). Our results suggest that metformin exhibits a protective effect, as evidenced by the decrease of oxidative stress in the brain.

### Metformin protected cognitive impairment following CLP

**Figure 3:**
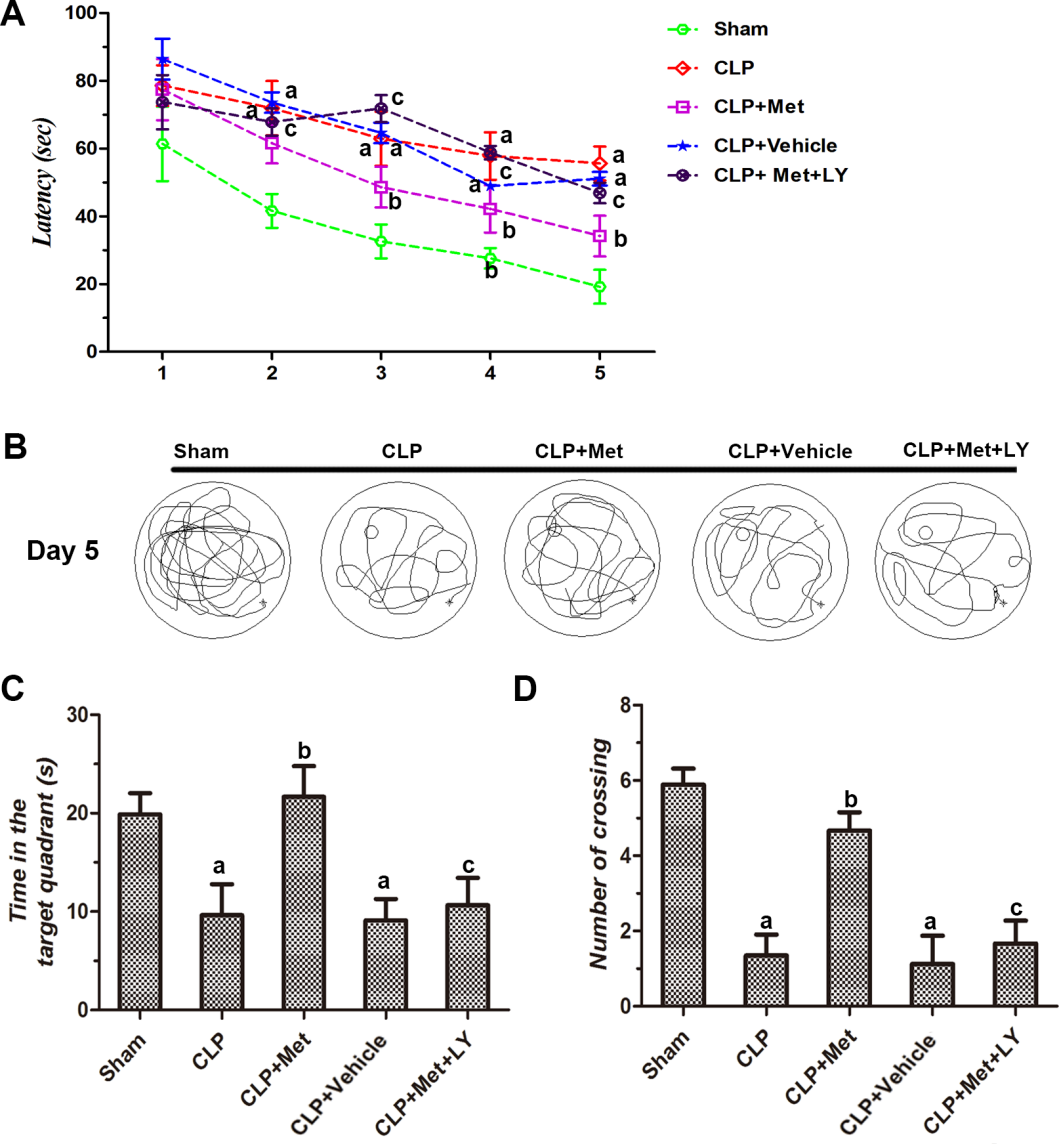
Effect of metformin on cognitive impairment in the MWM test (**A**) Escape latency in each group. (**B**) Track maps of mice in different groups on day 5 without platform. (**C**) Time in the target quadrant for different groups in the probe test. (**D**) Number of crossings of different groups in the probe test; data are presented as the mean ± S.E.M (*n* = 6); (A) *P* < 0.05 relative to the Sham group; (B) *P* < 0.05 relative to the CLP group; (C) *P* < 0.05 relative to the CLP+ Met group.

In our study, we used the MWM test to determine the effect of metformin on cognitive function. Throughout the training, the escape latency in all groups gradually decreased. However, in the CLP group, the escape latency was markedly longer than it was for the Sham group (*P <* 0.05, Figure 3A). While the metformin treatment significantly decreased the escape latency, the latency could be eradicated by an LY treatment (*P <* 0.05, Figure 3A). In the probe test, our results indicated an improvement in cognitive function in the metformin-treated septic mice compared with the CLP mice (*P <* 0.05, Figure 3B–3D). However, the time in the targeted quadrant and the number of platform location crosses were significantly decreased with the LY treatment compared with the CLP+ Met group (*P <* 0.05, Figure 3B–3D). Overall, our data indicate that metformin can protect mice from the cognitive impairment induced by CLP.

### Metformin decreased the neuronal loss after CLP

**Figure 4:**
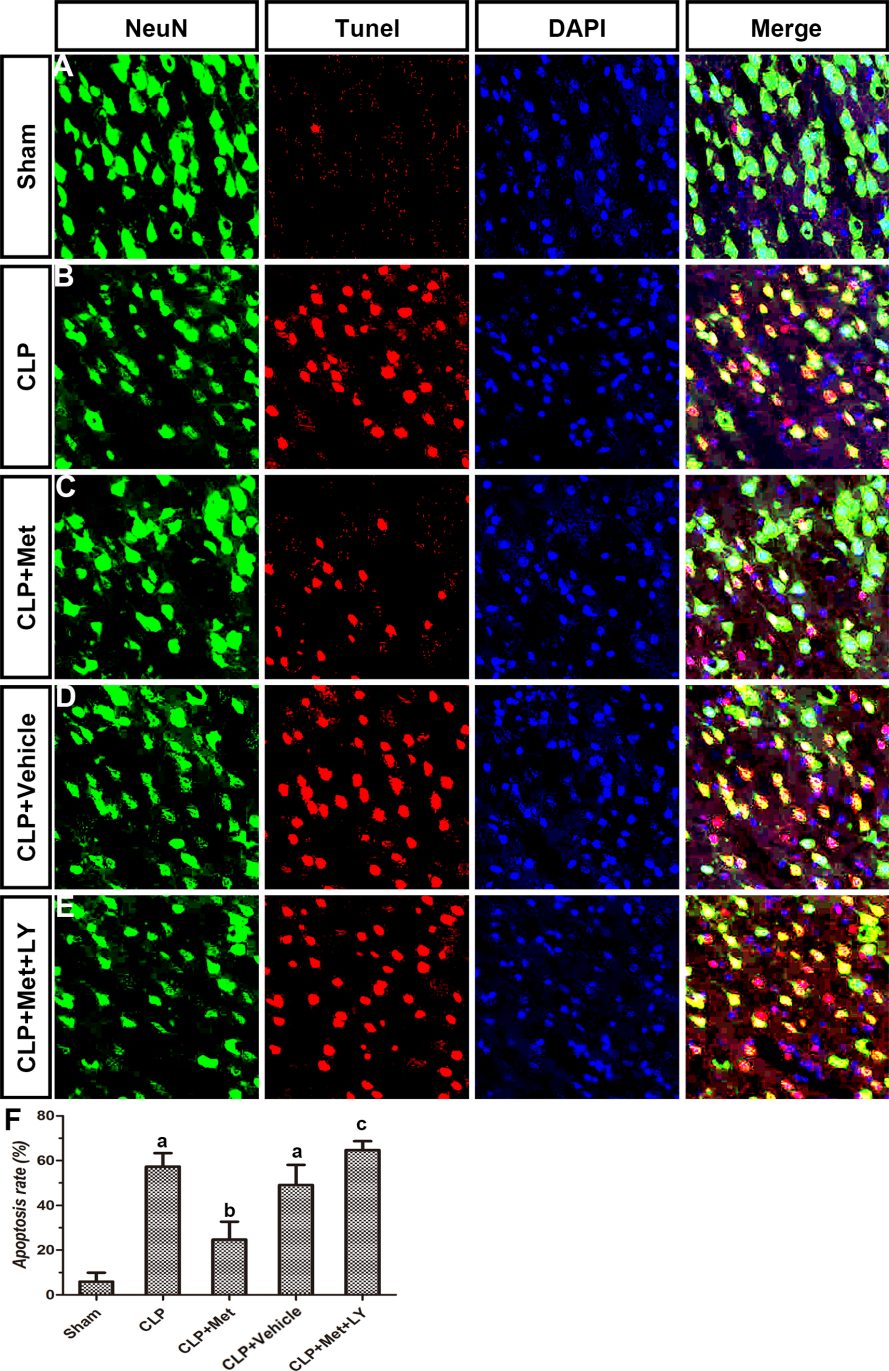
Effect of metformin on neuronal apoptosis in cerebral cortex using TUNEL assay and immunohistochemistry (**A**–**E**) Photographs of NeuN staining, TUNEL assay, DAPI staining and merge in each group. (**F**) Apoptosis rate in different groups; data are presented as the mean ± S.E.M (*n* = 6); (A) *P* < 0.05 relative to the Sham group; (B) *P* < 0.05 relative to the CLP group; (C) *P* < 0.05 relative to the CLP+ Met group.

**Figure 5:**
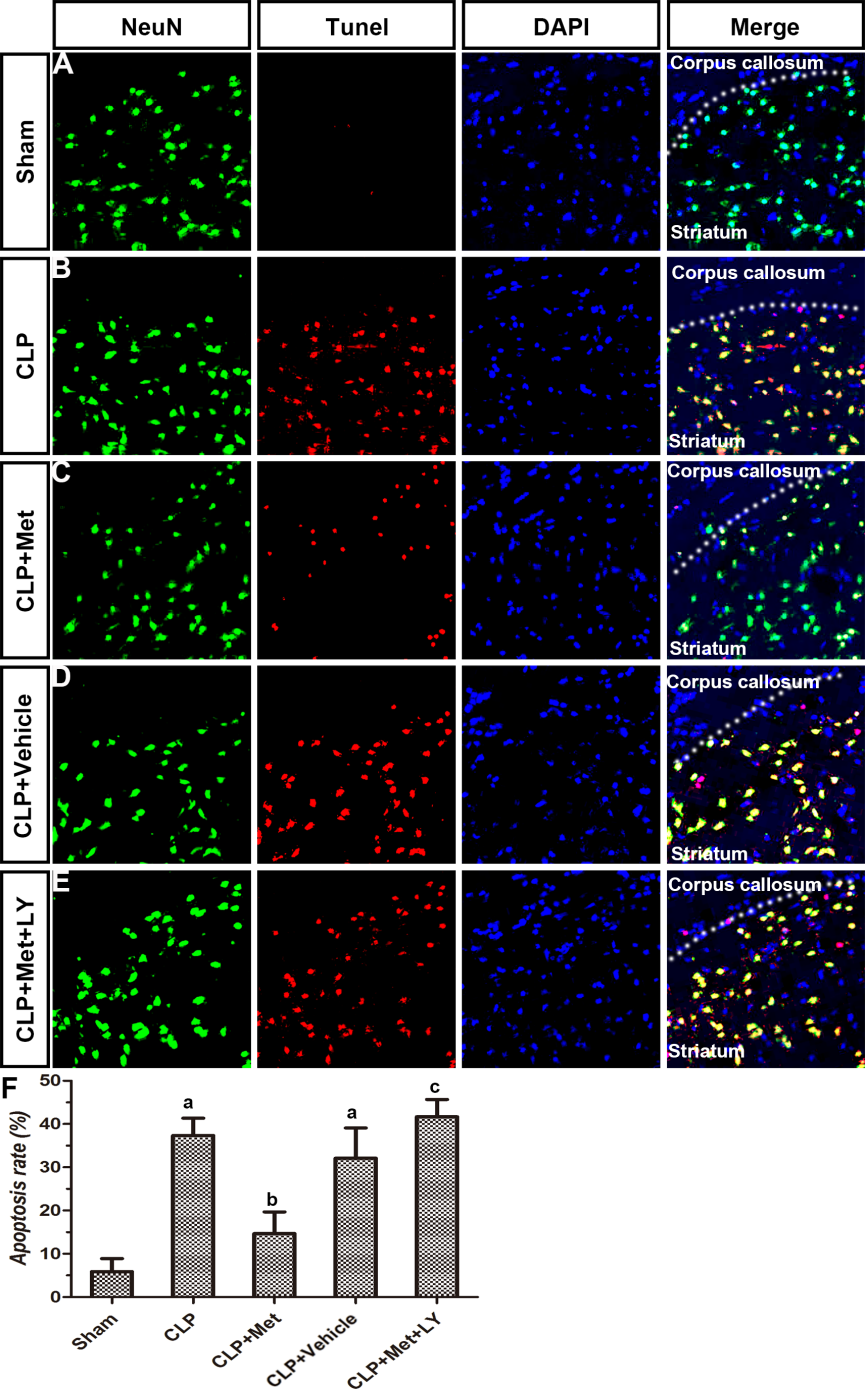
Effect of metformin on neuronal apoptosis in striatum using TUNEL assay and immunohistochemistry (**A**–**E**) Photographs of NeuN staining, TUNEL assay, DAPI staining and merge in each group. (**F**) Apoptosis rate in different groups; data are presented as the mean ± S.E.M (*n* = 6); (A) *P* < 0.05 relative to the Sham group; (B) *P* < 0.05 relative to the CLP group; (C) *P* < 0.05 relative to the CLP+ Met group.

**Figure 6:**
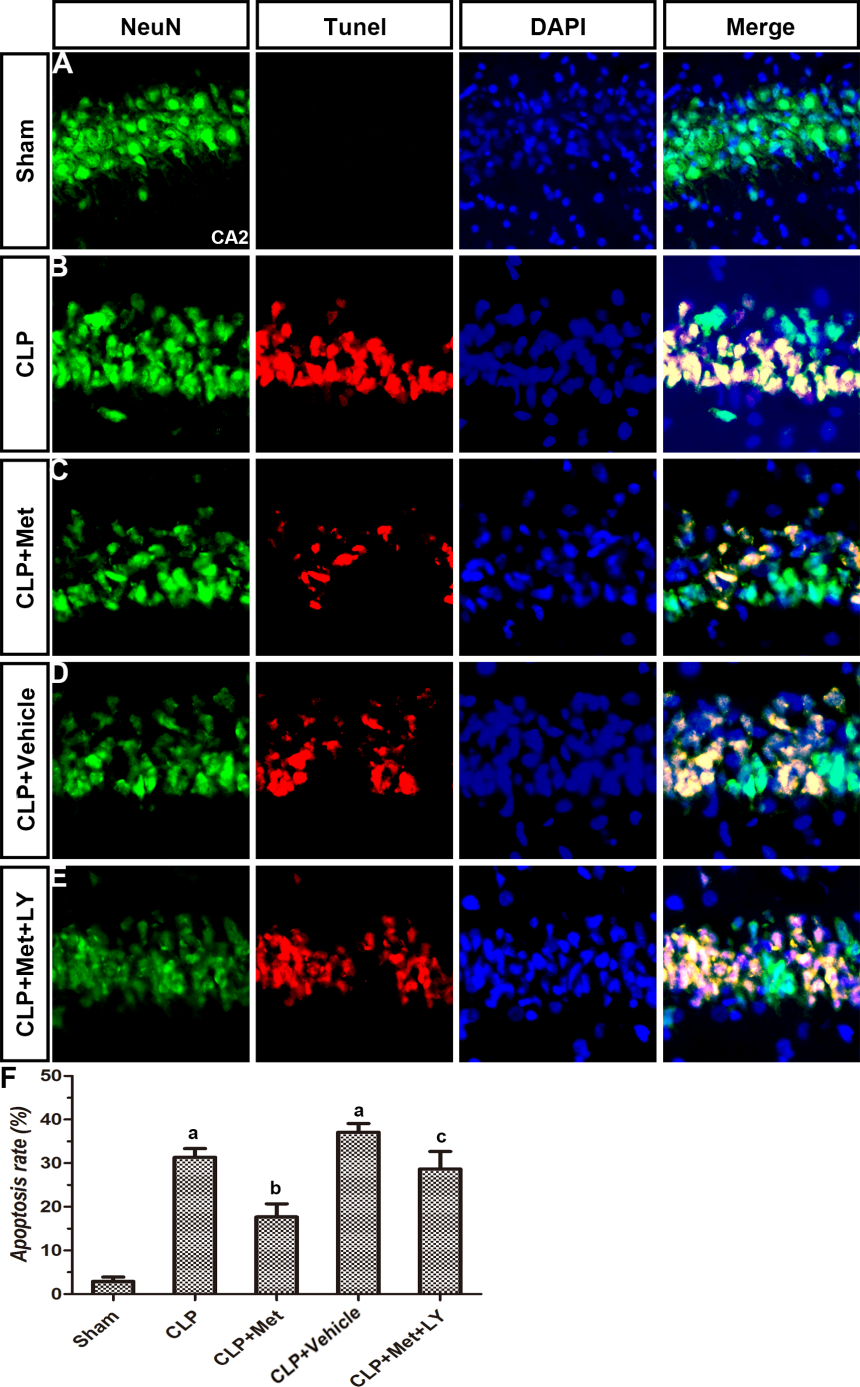
Effect of metformin on neuronal apoptosis in hippocampus CA2 using TUNEL assay and immunohistochemistry (**A**–**E**) Photographs of NeuN staining, TUNEL assay, DAPI staining and merge in each group. (**F**) Apoptosis rate in different groups. Data are presented as the mean ± S.E.M (*n* = 6); (A) *P* < 0.05 relative to the Sham group; (B) *P* < 0.05 relative to the CLP group; (C) *P* < 0.05 relative to the CLP+ Met group.

TUNEL assays and immunohistochemistry methods were applied to examine the protective effect of metformin. Compared to the Sham group, the CLP group exhibited extensive neuronal apoptosis in the cerebral cortex, but the administration of metformin resulted in decreased neuronal loss due to CLP (Figure 4). However, the effect of metformin can be eliminated with an LY treatment. The diagram illustrates that the rate of apoptosis increased after CLP, whereas the metformin treatment significantly decreased the rate of apoptosis in the CLP+ Met group (*P <* 0.05, Figure 4F). The number of neurons in the striatum and hippocampus also decreased after CLP, whereas the metformin treatment protected neuron loss induced by CLP. However, an LY treatment can reverse the effect of metformin (Figures 5, 6). These results suggest that metformin can partially reverse the neuronal loss in the cerebral cortex, striatum and hippocampus caused by CLP.

### Metformin increases the levels of p-Akt following CLP

**Figure 7:**
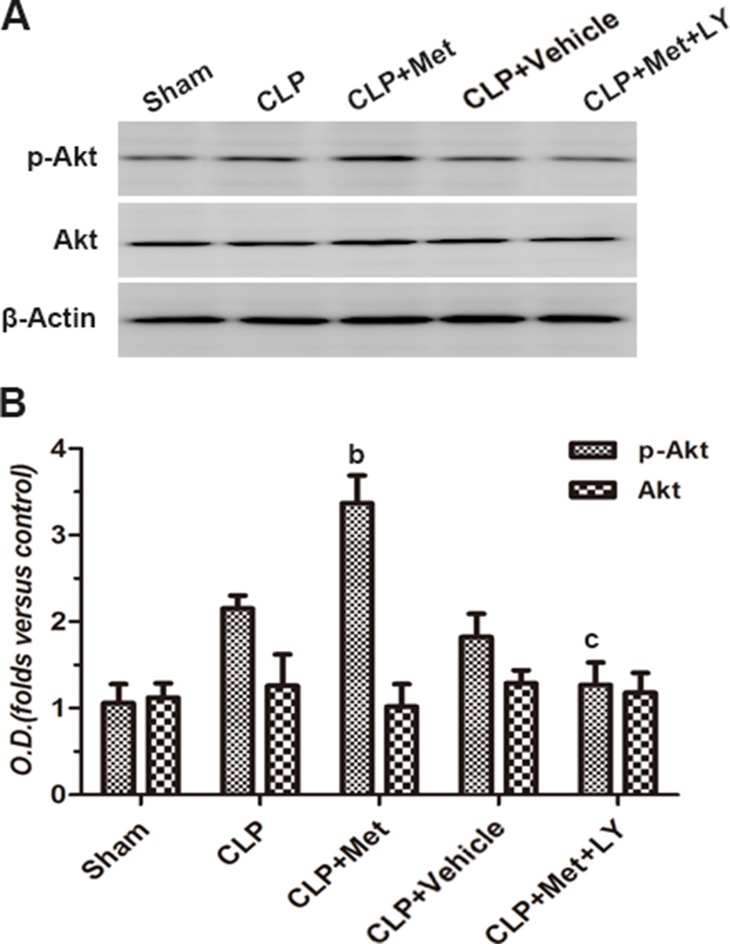
Effect of metformin on phosphorylation of Akt protein (**A**) Immunoblot bands in each group were shown. (**B**) The intensity of the bands is presented as optical density (O.D.) analysis. Data are presented as the mean ± S.E.M (*n* = 7); (A) *P <* 0.05 relative to sham group.

To further evaluate the mechanism underlying the protective effect of metformin against CLP, we conducted a western blot analysis to assess the levels of p-Akt. Although surgery could induce an increase in p-Akt levels, it had no statistical significance. Further, although metformin treatment in the CLP+ Met group markedly increased the levels of p-Akt compared to the CLP group, the phosphorylation of Akt could be eliminated by the administration of LY (*P <* 0.05, Figure 7). Moreover, there were no significant changes in the expression of total Akt. Nonetheless, the data indicate that metformin can enhance the amount of Akt phosphorylation and thereby produce a protective effect.

## DISCUSSION

In our study, we found that metformin can protect mice brains from sepsis via the anti-inflammatory effect, the anti-oxidative effect and the increase of p-Akt, which further decreases neuronal loss in the cerebral cortex, striatum and hippocampus. This protective effect, however, can be eliminated by the administration of LY294002, a PIK3 inhibitor, which indicates that metformin acts by activating the PI3K/Akt signaling pathway.

Cecal ligation and puncture result in severe mortality, increased brain water content and the breakdown of BBB. This model could be used to mimic the septic patients in the clinic. Our data indicate that brain water homeostasis is disrupted as a result of sepsis. Moreover, SAE is a severe complication of sepsis with no clear pathogenesis, and its clinical manifestation includes confusion, delirium and coma [1]. Many patients with SAE suffer from long-term cognitive impairment, progressive immunosuppression and anti-inflammatory deficiency [22]. The proinflammatory cytokines, such as TNF-α and IL-1β, accumulate in the plasma. Once inside the brain, the proinflammatory cytokines interact with endothelial cells and microglia to produce more cytokines and ROS, which results in further aggravated brain injury [23, 24]. In our study, we examined the levels of inflammatory cytokines in serum and found that the levels of IL-6, IL-1β and TNF-α were dramatically increased after CLP, consistent with previous studies [25, 26]. Oxidative stress is also a pivotal factor in triggering brain injuries from sepsis [27]. The substantial production of a large number of ROS, which are beyond elimination, can induce lipid peroxidation, damage to membranes of the cell and mitochondria and neuronal apoptosis [28]. Moreover, a previous study described that because oxidative central nervous system damage occurs primarily in the hippocampus, it is associated with cognitive impairment [29]. SOD is a key element in clearing ROS from our body and can thus prevent the damage caused by ROS. MDA, which is a direct product of lipid peroxidation, is a well-used indicator of the degree of lipid peroxidation. Our results provide evidence that metformin can exert an anti-inflammatory effect and an anti-oxidative effect in the brain.

A previous study demonstrated that sepsis-induced oxidative damage was present in the hippocampus of the rodent at 6 h post CLP, and the pharmacological method to prevent oxidative changes in the hippocampus attenuated learning and memory impairment after CLP [13, 29, 30]. To investigate the effect of metformin in cognitive function after sepsis, we performed the MWM test. Our results indicated that metformin rescued the cognitive dysfunction induced by CLP. We then checked the neuron apoptosis in the brain region of the cerebral cortex, striatum and hippocampus, all of which are closely related to the sepsis-induced brain damage. As evidenced by our data, the metformin treatment decreased the neuronal apoptosis caused by CLP.

The PI3K/Akt signaling pathway has been reported to play a pivotal role in sepsis [25]. PIK3 activation leads to Akt phosphorylation, which is further involved in diverse molecular cascade reaction. The phosphorylation of Akt can preserve mitochondrial integrity and promote cell survival against the inflammation and oxidative stress induced damage [31]. A series of studies has determined that metformin can modulate the PI3K/Akt signaling pathway. Furthermore, metformin can attenuate damage such as hepatic insulin resistance by improving the PI3K/Akt signaling pathway [32, 33]. Our data suggest that the administration of metformin significantly increases the levels of p-Akt levels and that the protective effect of metformin on anti-inflammation, anti-oxidation, cognitive protection, anti-apoptosis as well as the increase in p-Akt are eradicated by LY294002. Taken together, our results suggest that the PI3K/Akt signaling pathway is involved in the protective effect of metformin against sepsis-induced brain injury.

In summary, metformin treatment alleviates brain damage after sepsis. Metformin can inhibit an inflammatory response, relieve oxidative stress and promote p-Akt production, which further prevents neuronal apoptosis in the cerebral cortex, striatum and hippocampus. Accordingly, our study provides evidence for the future clinical use of metformin in treating patients suffering from sepsis.

## MATERIALS AND METHODS

### Animals

Male C57/BL6 mice were obtained from the Shanghai Experimental Animal Center (Chinese Academy of Science, Shanghai, China). All mice were housed under a pathogen-free condition at 25 ± 1°C, with free access to water and food. The home cages were maintained in 12-h light/12-h dark cycles. All experiments were performed on healthy adult mice weighing 22 to 25 g. The present study was approved by the University Ethics Committee for Animal Experimentation.

### Drugs and antibodies

The following drugs and antibodies were applied: metformin (M0605000), LY294002 (L9908), Evans blue (E2129), DAPI (D8417) and Alexa Fluor 488 goat-anti-rabbit secondary antiserum (SAB4600234) obtained from Sigma (USA). Monoclonal anti-NeuN primary antiserum was obtained from Merck Millipore (Darmstadt, Germany). Chromo 546-conjugated anti mouse-IgG, horseradish peroxidase-conjugated anti-rabbit and anti-mouse IgG were purchased from Abcam (Cambridge, MA, USA). Akt, phospho-Akt and β-actin were obtained from Chemicon.

### Experimental design

All mice were randomly assigned to one of five groups: the Sham group (*n* = 60), CLP group (*n* = 60), CLP+ Met group (*n* = 60), CLP+ vehicle group (*n* = 60), CLP+ Met+ LY group (*n* = 60). Mice were administered metformin once a day for 11 days (four days before surgery and seven days after surgery) and LY one hour before CLP. Approximately 24 h after CLP, six mice were sacrificed to perform ELISA and lipid peroxidation and antioxidant enzyme assays. Approximately 48 h after CLP, six mice were sacrificed to measure brain water content, six mice were sacrificed to measure blood-brain barrier permeability assay and seven mice were used to perform a western blot assay. Seven days after CLP, mice performed the Morris water maze test, the TUNEL assay and the immunohistochemistry assay.

### Groups and CLP model

The CLP model was established according to previous studies [19]. In brief, mice were anesthetized with an intraperitoneal injection of 50 mg/kg pentobarbital sodium and subsequently positioned on a sterilized operating table. A 2-cm abdominal midline incision was made to expose and mobilize the cecum. Colon contents were lightly squeezed to fill the cecum, which was ligated distal to the ileocecal valve to maintain intestinal continuity. A 20-gauge needle was punctured once through the anterior and posterior walls of the cecum. Subsequently, a small amount of fecal content was squeezed through the puncture site. The bowel was returned to the peritoneal cavity and the incision was sutured with a sterile 4–0 silk. The same surgery, although without CLP, was performed on the Sham-operated mice. Mice in all groups received 0.9% sterile normal saline subcutaneously (5 ml/100 g body weight) to resuscitate them after surgery. Following surgery, the mice were returned to the homecages and observed until subsequent experiments. Metformin was dissolved in normal saline and injected into mice intraperitoneally at 100 mg/kg in the CLP+ Met group and CLP+ Met+ LY group. Mice in the CLP+ vehicle group received equal volumes of normal saline intraperitoneally. One hour before CLP, 10μl of LY solution (50 mmol/L dissolved in 25% DMSO in phosphate-buffered saline [PBS]) was injected into the left ventricle ((bregma; 1.0 mm lateral, 0.3 mm posterior, 2.6 mm deep) at a rate of 1 μL/min in the CLP+ Met+ LY group. Other groups received an equal amount of normal saline.

### Evaluation of survival percentage

The mice in each group were raised in pathogen-free conditions with free access to water and food. The survival percentage was measured within 7 days after surgery.

### Brain water content

A dry-wet weight method was used to measure brain water content [20]. Approximately 48 h after surgery, the mice were anesthetized and sacrificed and the brains were removed. Brain samples were immediately weighed to obtain the wet weight. Next, the brain samples were dried at 100*°*C for 24 h and reweighed to obtain the dry weight. The brain water content was calculated as (wet weight–dry weight)/wet weight × 100%.

### ELISA

We measured the levels of IL-6, IL-1β and TNF-α in the hippocampus. Approximately 24 h after surgery, the mice were sacrificed, and their brains were quickly removed. In an ice-cold environment, the hippocampus was quickly isolated and stored at −80°C until use. The levels of IL-6, IL-1β and TNF-α were quantified using specific ELISA kits (Minneapolis, MN, USA) according to the manufacturer's instructions. The absorbance values were analyzed at 450 nm using a microplate reader (Tecan Group AG, Mannedorf, Switzerland) according to the color reaction.

### Lipid peroxidation and antioxidant enzyme assays

Samples stored at −80°C were used to measure the activities of SOD and the content of MDA. The assays were conducted according to manufacturer instruction using kits purchased from the Nanjing Jiancheng Company (A001, Nanjing, China).

### Morris water maze testing (MWM)

All mice were subjected to MWM testing to evaluate learning and memory ability [21]. In brief, mice underwent 3 swimming trials per day for 4 consecutive days to strengthen their memory. While the platform was kept in the same position throughout the test, the starting position of each trial was chosen randomly with the heads of the mice facing the wall. The trials were automatically terminated once the mice reached the platform or 120 s had elapsed. If the mice failed to reach the platform in 120 s, they were gently guided to the platform. The mice were allowed to remain on the platform for 20 s before starting a new trial. The time that each mouse spent on finding the platform was measured as the escape latency, and anything over 120 s was recorded as 120 s. The hidden platform was removed on the day of the experiment. Next, mice were placed in the place farthest from the platform and allowed to swim for 120 s. The latency, time in the target quadrant and number of crossings were measured. The MWM behavior was analyzed using the ANY-maze video tracking system (Stoelting, Wood Dale, IL, USA) with a CCD camera.

### Blood-brain barrier (BBB) permeability assay

Evans blue (EB) dye was used to examine the BBB permeability. Briefly, the mice were injected with EB (3 ml/kg, 2% in normal saline) through the tail vein at 48 h after CLP surgery. After 2 h of circulation, the mice were anesthetized and sacrificed. Their brains were quickly removed, weighed and incubated in formamide (1 ml) for 48 h. Next, the supernatant was collected after centrifugation, and the values were measured at OD 623 nm using a microplate reader (Multiskan Spectrum, Thermo Scientific, USA). The amount of EB (μg/g) was quantified according to a linear standard curve between optical density and concentration.

### TUNEL assay

Neuronal apoptosis was analyzed using terminal deoxynucleotidyl transferase dUTP nick end labeling (TUNEL) assay. Briefly, the mice were anesthetized and perfused with 25 ml 0.9% normal saline, followed by a 25 ml 4% paraformaldehyde infusion. Brains were isolated and placed in 4% paraformaldehyde to post-fix for 24 h. Next, brains were dehydrated and dissected at a thickness of 16 μm. An *In Situ* Cell Death Detection Kit (TMR Green; Roche Applied Sciences, Indianapolis, IN) was used to determine the number of apoptotic cells. TUNEL+ nuclei were visualized on the confocal microscope and were quantified using ImageJ.

### Immunohistochemistry

The sections were blocked with 5% goat serum for 1 h. After being washed with a PBS, a monoclonal anti-NeuN primary antiserum, i.e., mouse Cy3-conjugated anti-NeuN, 1: 100, was incubated overnight, followed by incubation with chromo 546-conjugated anti mouse-IgG (1: 500;). DAPI 1:1000 was then incubated for 5 min. The NeuN-ir and DAPI-ir nuclei were visualized using confocal microscopy.

### Western blot analysis

Brain cortex samples were collected and protein extractions were obtained 48 h after surgery. Total protein concentration was measured using a bicinchoninic acid (BCA) protein assay kit (Beytime, Haimen, China). The protein was separated using 10% SDS-PAGE gels and electro-transferred to polyvinylidenedifluoride (PVDF) membranes. The membranes were blocked with 5% skim milk in TBST at room temperature for 2 h and then incubated with primary antibodies against Akt, phospho-Akt and β-actin (1:1000) at 4*°*C *overnight*. Subsequently, the membranes were washed and incubated with secondary antibodies (1:5000) at room temperature for 1.5 h and washed three times with TBST. The density of the bands was visualized with X-ray film (Kodak, Shanghai, China) and quantified using an image analyzer (LabWorks Software, Upland, CA).

### Statistical analysis

The data of each experiment were presented as the mean ± S.E.M., and GraphPad Prism 6.0 software was used to analyze the statistics. The Fisher exact test probability method was applied to analyze the survival rates, and statistical significance was conducted via a one-way ANOVA followed by the Newman–Keuls test. A probability less than 0.05 was the criterion for statistical significance.

### Abbreviations

CLP: cecal ligation and puncture; Met: metformin; LY: LY294002; BBB: alteration of blood-brain barrier; SAE: sepsis associated encephalopathy; PI3K: phosphatidylinositol-3-kinase; MWM: Morris water maze testing; EB: Evans blue; TUNEL: terminal deoxynucleotidyl transferase dUTP nick end labeling.
